# The Application of Collaborative Business Intelligence Technology in the Hospital SPD Logistics Management Model

**Published:** 2017-06

**Authors:** Tongzhu LIU, Aizong SHEN, Xiaojian HU, Guixian TONG, Wei GU

**Affiliations:** 1. School of Management, Hefei University of Technology, Hefei, PR China; 2. Division of Medical Engineering, Anhui Provincial Hospital, Hefei, PR China

**Keywords:** Business intelligence, SPD model, Logistics management, Hospital

## Abstract

**Background:**

We aimed to apply collaborative business intelligence (BI) system to hospital supply, processing and distribution (SPD) logistics management model.

**Methods:**

We searched Engineering Village database, China National Knowledge Infrastructure (CNKI) and Google for articles (Published from 2011 to 2016), books, Web pages, etc., to understand SPD and BI related theories and recent research status. For the application of collaborative BI technology in the hospital SPD logistics management model, we realized this by leveraging data mining techniques to discover knowledge from complex data and collaborative techniques to improve the theories of business process.

**Results:**

For the application of BI system, we: (i) proposed a layered structure of collaborative BI system for intelligent management in hospital logistics; (ii) built data warehouse for the collaborative BI system; (iii) improved data mining techniques such as supporting vector machines (SVM) and swarm intelligence firefly algorithm to solve key problems in hospital logistics collaborative BI system; (iv) researched the collaborative techniques oriented to data and business process optimization to improve the business processes of hospital logistics management.

**Conclusion:**

Proper combination of SPD model and BI system will improve the management of logistics in the hospitals. The successful implementation of the study requires: (i) to innovate and improve the traditional SPD model and make appropriate implement plans and schedules for the application of BI system according to the actual situations of hospitals; (ii) the collaborative participation of internal departments in hospital including the department of information, logistics, nursing, medical and financial; (iii) timely response of external suppliers.

## Introduction

Hospital logistics is one of the application fields for logistics. It mainly covers the procurement, storage, distribution, usage and the control of medical equipment, drugs and supplies in hospital. Since 1950s, scholars have paid attention to hospital material management, especially in the inventory management of medical materials. With the development of supply chain and logistics related theories, the hospital logistics attracted extensive attention, and many scholars started to systematically explore the hospital logistics (1–3).

Their primarily focus was on the development of management models in the field of procurement, inventory and distribution.

In recent years, several models have been proposed by scholars in terms of procurement. Lapierre et al. (4) designed procurement decision-making method of hospital materials with the tabu search algorithm. By analyzing the collaborative supply model between hospitals and medical suppliers, Centobelli et al. (5) proposed a more streamlined procurement strategy of medical supplies based on e-business. With the help of fuzzy failure mode and effect analysis (FMEA), Kumru et al. (6) improved the procurement procedures and methods. When it comes to the inventory management, Bijvank et al. (7) tried to optimize the hospital inventory by designing and using the capacity model and ability model. Shan et al. (8) employed the method of greedy algorithm to deal with the multi-level inventory problems in the hospital. Other researchers (9–11), proposed selecting the optimal distribution model with the methods of process modeling and mixed linear programming modeling based on the graph theory.

In some developed countries, several hospitals have adopted a new type of logistics management model, which is called SPD (supply, processing and distribution) model (12). The SPD model contains three links in the hospital logistics supply chain including supply, processing and distribution. In 1960s, Gordon A. Friesen, firstly put forward the idea of “hospital logistics management and supply integration”. The original purposes of the idea were to realize the integration management of procurement, inventory, distribution and consumption with the support of informationization. With the recent development in this field, SPD model has been partly applied to hospitals’ daily operation and management in several countries including Japan and China (12–15). In Japan, the SPD model has realized the unified management of hospital logistics procedures such as procurement, usage, recovery and distribution of medical products (e.g., drugs, medical supplies and equipments) with the help of information systems. In China, several hospitals in Shanghai, Nanjing, Tianjing and Lhasa, have introduced the SPD model, in which the procurement, inventory, package and distribution of hospital supplies were entrusted to a third-party company, and hospitals only need to pay for the supplies according to the actual consumption. Although there are many advantages of SPD model in hospital logistics management, the intelligence and collaboration of SPD model are still relatively low, which hindered the improvement of management efficiency to large extent.

Business intelligence (BI) transfers various data from enterprises into information or knowledge displayed in a cooperative manner that enterprise managers interested in. They were able to provide scientific evidence for the enterprise managers’ decision-making and finally strengthen the competitive advantage of enterprises. In the early stage, scholars primarily focused on the data warehouse and data marts. Ariyachandra et al. (16) put forward the architecture of data mart bus and selected the data mart structure of consistent correlation dimension. One year later, they also proposed an independent and separated architecture for the data mart (17). Schaffner et al. (18) proposed hub-and-spoke architecture and chose centralized data warehouse as well as dependent architecture of data mart. Wu et al. (19) suggested the architecture of SOA-ITPA and adopted service-oriented reusable component technology as well as extensive ETL (extract, transform and load) services, in which the components communicate with each other via open standard message protocol such as XML and SOAP. However, with the change of business environment, the existing BI systems can hardly meet the demands of efficient data analysis and collaborative business process optimization.

With the development of hospitals in terms of construction scale, structure complication and management informationization, the need for scientific, standardized and lean management of hospitals is more obvious than ever. As one of the important aspects in hospital management, logistics management is now facing the realistic problems of low efficiency and high cost.

This study aimed to explore the key technologies of hospital logistics collaborative BI system including architecture, data warehouse, data mining and collaboration, so as to improve the efficiency of hospital SPD logistics management model.

## Materials and Methods

We performed literature review to understand SPD and BI related theories and recent research status. Firstly, we searched Engineering Village database for English articles and China National Knowledge Infrastructure (CNKI) for Chinese articles published from 2011 to 2016. In addition, we searched Google for related books, conference papers, Webpages, etc. The keywords for literature review included “SPD”, “supply, processing and distribution”, “BI”, “ business intelligence”, “hospital”, “logistics”, and “supply chain”. At last, we incorporated the ideas from the literature review into a practical study protocol of the application of collaborative BI technology in the hospital SPD logistics management model.

**Fig. 1:**
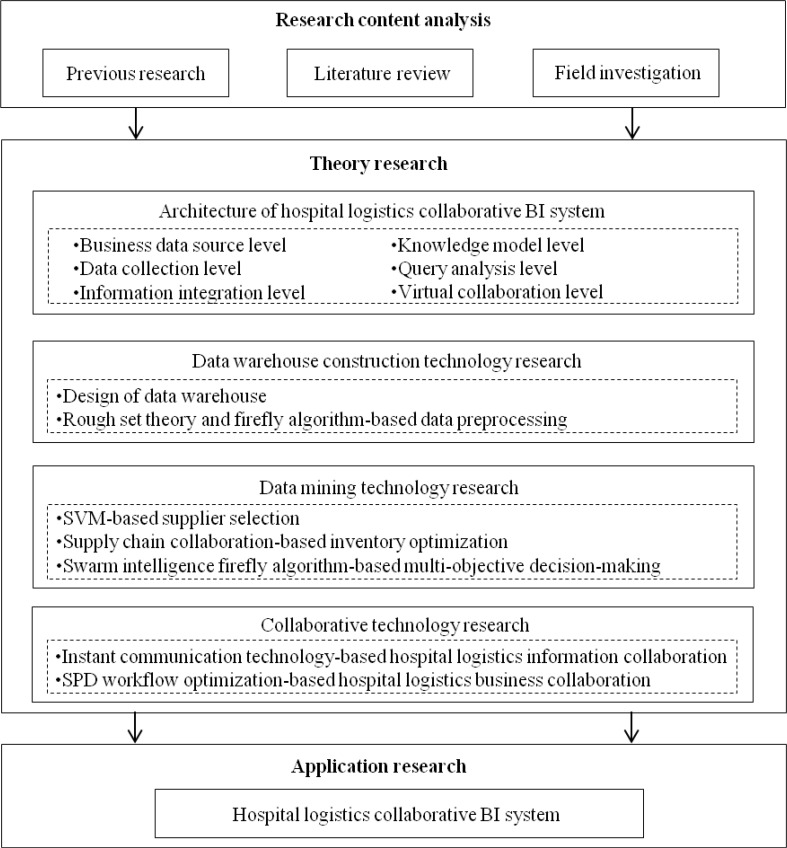
The overall technology roadmap of the study (Note: BI=Business intelligence)

In this study, several technologies and methods were employed. Specifically, we adopted the methods of rough set theory and firefly algorithm-based data preprocessing to construct the data warehouse, we utilized improved data mining technologies including support vector machine (SVM) supplier selection, supply chain collaboration-based inventory optimization and swarm intelligence firefly algorithm-based multi-objective decision-making to solve key problems in hospital logistics collaborative BI system, we used instant communication technology and SPD workflow optimization method to realize the collaboration of BI system, and we selected Windows Vista operating system as the development environment of experimental platform. The overall research technology roadmap of study is presented in Fig.1.

## Results

### Architecture of hospital logistics collaborative BI system

**Fig. 2:**
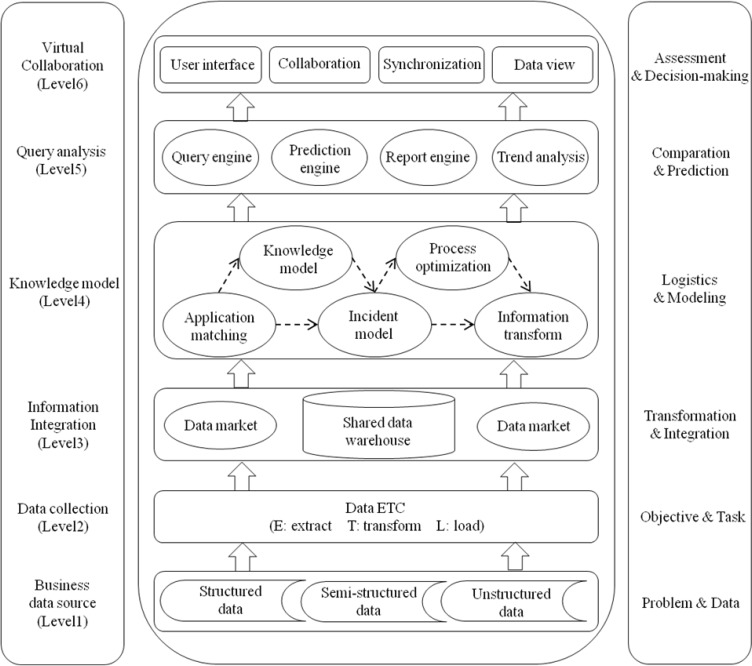
The architecture of collaborative business intelligence for hospital logistics

Collaborative BI combines the traditional BI and collaborative technology to satisfy the specific needs of business enterprises’ collaborative decision-making and business process. Hospital logistics collaborative BI system is divided into 6 levels including business data source, data collection, information integration, knowledge model, query analysis and virtual collaboration:

Level 1: Business data source level refers to structured, semi-structured and unstructured source data provided by different main bodies of hospital logistics supply chains and internal departments of hospital. They are original source data and problems on the hospital logistics supply chain.
Level 2: Data collection level provides connect and access to a variety of hospital logistics operation management related data source. It maps various data sources to the handler to realize the data extraction, transformation and update.
Level 3: Information integration level is the level that integrates many of the underlying data sources. It uses the source data from data warehouse to describe data extracted from data collection level and reshape the data syntax and semantics of the next level, to realize the construction of shared data warehouse and complete the multi-source data transformation and integration of hospital logistics supply chain.
Level 4: Knowledge model level describes the business processes, knowledge and information models of hospital logistics management, and the semantics regarding their interdependence as well as correlation. Besides, it will modeling based on the relationships between shaped knowledge models extracted from the perspective of business processes and users. This way, it would realize information and business collaborative process logic modeling between different main bodies of hospital logistics supply chain.
Level 5: Query analysis level provides tools and applications, which are considered the cores of the collaborative BI function. These include query, report, correlation analysis, trend and predictive analysis. These functions can be called by virtual collaboration level to set up the views and reports, to learn and perform model analysis, as well as to provide visualized exploration of real-time data to facilitate comparison and prediction of final information.
Level 6: Virtual collaboration level provides a rich user interface and virtual spaces based on logistics business specific parameter setting, which are employed to display and solve problems in hospital logistics including medical consumables supplier selection, inventory optimization, product distribution, information coordination, and business coordination, etc. These facilitate users to check, share, analyze, and coordinate different forms of data, and finally to realize the users’ assessment and decision-making (Fig. 2).

The architecture mainly consists of 7 function modules, these include multi-source heterogeneous data preprocessing module, shared data warehouse construction module, SVM-based medical consumable materials supplier classification service module, swarm intelligence firefly algorithm-based inventory optimization module, swarm intelligence firefly algorithm-based medical consumables distribution module, information collaboration service module and business collaboration service module. System management is composed of operation maintenance management and security management.

### Data warehouse construction technology research

The construction of data warehouse used centralized storage, retrieval, and other corresponding multi-source data process in the environment of collaborative BI. At the same time, rough set data mining algorithm and swarm intelligence firefly optimization algorithm were also employed to deal with the data in the data warehouse. These provided reliable data for data analysis services of collaborative BI system, and to improve accuracy and efficiency of data analysis services.

### Design of data warehouse

Shared data warehouse can be divided into two types including centralized and decentralized models. The centralized shared data warehouse requires creating physical data warehouse at first. The storage and management of data in centralized shared data warehouse is operational data that has been processed and arranged. When decisions are made, every related enterprises and internal departments transmit data to the data warehouse via specified data marts. This made centralized data warehouse to have some extent of advantages in keeping the consistency of data. By contrast, decentralized data warehouse is set up based on virtual overall data warehouse. Our study aimed to establish a centralized shared data warehouse via the integration of data marts in hospital logistics management, in order to realize the multi-agent data support in the collaborative BI system and satisfy the collaborative BI system users’ demands of data analyses and data-mining of specified business information.

There were many unstructured or semi-structured data in different parts of the hospital logistics supply chain including the evaluation information of suppliers, the qualifications of medical manufacturers, as well as document information for hospital internal communications. In order to better deal with these types of data we first created corresponding document template according to specific application needs and subsequently tried to write transform programs to read the contents of these unstructured/semi-structured files and adopted different rules to transform them to standard XML documents. Finally, we analyzed the XML documents and relational database and figured out some rules for the transformation from XML documents to relational database tables. This was supported by the relational model-based data warehouse. The construction of data warehouse in hospital logistics BI system was the standardized integration of information flows from upstream to downstream of logistics supply chain. Considering the fact that the flow of information upstream was heterogeneous and the structure of raw data varied across different parts of supply chain, information systems, as well as modules, we took several measures to define the metadata uniformly. At first, according to the Berlin core data set, we provided metadata information for every member in the supply chain by reference to uniformed metadata specifications that were defined by the logistics alliance. Secondly, data exchange and integration among various systems were achieved through metadata mapping. Finally, according to the idea of oriented to application integration (OAI), we designed a metadata framework and information exchange model from the perspective of logistics information exchange to realize the data integration of different parts in the hospital logistics supply chain.

Data storage refers to the storage mechanism of shared data warehouse in hospital logistics collaborative BI system. In order to solve the existing contradiction between model stability and demand variability, we stratified the data storage of shared data warehouse into three levels: (i) temporary buffer area, (ii) integrated data area and (iii) summary data area. The buffer function loads multi-source system data in hospital logistics supply chain into a temporary buffer area, the area works in the course of transform and load from data source system to integrated data area. Integrated data area, the core of the whole data storage, analyses every event from different dimensions via constructing multi-dimensional model. The integrated data area can be divided into three sub-areas: (i) operational data area, (ii) data analysis area and (iii) data archiving area. The operational data area stores data that were classified according to the application themes and provides uniformed hospital logistics data views based on real-time operation data. The data analysis area mainly stores historical data of hospital logistics operation decision analysis. The data archiving area is used to store the historical archived data derived from operational data area and data analysis area. The summary data area is a virtual zone without actual stored data, it is arranged to store the extra-preprocessed data for the use of the font-end. According to the relevant definition in the database, the data are named with clear business meaning, i.e., integrate the tables, fields and their complicated relationships into business terms or indicator names that could directly be displayed in the font-end.

### Rough set theory and firefly algorithm-based data preprocessing

In order to improve the efficiency and accuracy of swarm intelligence algorithm-based data mining, we used a rough set theory to perform data preprocessing of data warehouse. The data mining technology combined the rough set theory and firefly algorithm together, which not only took the quality of sub-data sets into consideration, but also ensured the subtraction of raw data to the maximum extent. The objective function of the firefly algorithm is: $\mathrm{Fitness}\;(X)=\frac{m-\left|X\right|}{m}+\frac{n\left|R\right|\gamma_{X}(D)}{m\Gamma }$ ; m=|c|, refers to the length of a firefly; *γ*_x_*(D)* refers to the quality of data sets; R is the subtraction of C (condition attribute). If the decision tables are continuous, $\Gamma =\left|\gamma_{1}\right|+\left|\gamma_{2}\right|$ , or else $\Gamma =\left|\gamma_{1}\right|$ .

The basic steps are as follows: at first, we identified a proper method of subtraction (R), depending on which firefly populations were randomly generated by coding. Then each individual was evaluated by fitness function that has already been designed. At last, we figured out subsets that met the function *γ*_s_*(D)* = *γ_c_(D)* by iteration. These steps helped us not only get the subsets with the smallest data scale, but also keep the original characters of data sets at the maximum extent.

### Data mining technology research

The data of collaborative BI system in the hospital SPD model is complex, multidimensional, and nonlinear. Using data mining, we were able to solve several problems including medical consumables supplier selection, inventory optimization, as well as medical consumables distribution. These were very important in the hospital logistics management. In other words, by adopting improved data mining algorithm that met the demand of collaborative BI system in the hospital SPD model, we achieved the following goals: (i) we realized more accurate and effective classification and selection of medical consumables suppliers; (ii) we completed the procurement control, inventory optimization and inventory shortage warning in the first-level warehouse of the SPD model; (iii) we solved the optimization problems associated with medical consumables distribution in the secondary consumption points of the SPD model, especially the problems associated with the dynamic adjustment of distribution point, period and amount.

### SVM-based supplier selection

The SVM method is widely used in the system evaluation. By mapping the points in low-dimensional space to high-dimensional space, we used the principle of linear partition to determine the classification boundaries. We attempted to design medical consumables supplier evaluation and classification methods based on SVM, to deal with specific problems of supplier selection in the hospital SPD model. We, first, extracted the objective function and constraint condition of medical consumables supplier selection in the hospital SPD model. Then we extracted feature data sets as the training sample source according to supplier multidimensional attribute to complete the design of classifier. Finally, we attempted to realize the actual classification of medical consumables suppliers by classifying the medical consumables supplier sample into corresponding categories via the already designed classifier.

### Supply chain collaboration-based inventory optimization

The core of the supply chain inventory optimization in hospital SPD logistics model is the effective control of first-stage warehouse inventory level. Given the close association existed among inventory level setting and procurement strategy and safe inventory, the amount of procurement and procurement period directly affects the level of inventory. Therefore, hospital logistics inventory optimization is considered a type of data-driven analysis and design task, which requires analysis on large amounts of data followed by establishing a relational model between inventory costs and service levels. According to the two-stage inventory structure design, we divided the inventory optimization method into two steps: (i) we figured out the key parameters’ approximate fluctuation scopes of first-stage warehouse inventory in the SPD model using the time series method; (ii) we designed discrete control parameters and construct corresponding solution spaces, and then identified the most feasible inventory optimization solutions by using swarm intelligence firefly algorithm.

### Swarm intelligence firefly algorithm-based multi-objective decision-making

The effect of medical consumables distribution in SPD model mainly depends on the satisfaction of multiple targets including distribution amount, distribution point, distribution lead time, as well as dynamic adjustment of the distribution amount. Considering the medical consumables distribution multi-objective decision-making in the model of SPD may face several conditions such as large data scale, complicate structure and dynamic objective, we employed the swarm intelligence firefly algorithm method to deal with the multi-objective decision-making problems. We, first, established a mathematical model of multi-objective decision-making via extracting multiple decision-making objectives such as timeliness, accuracy and economy. The construction of constraint condition set was then analyzed. Secondly, we identified the solution spaces of multi-objective decision-making using swarm intelligence firefly algorithm method. Finally, we periodically used swarm intelligence firefly algorithm to deal with dynamic data in the data warehouse, and to provide dynamic adjustment strategy for distribution amount, point, and lead time, as well as distribution amount adjustment.

### Collaborative technology research

Collaborative technology refers to all types of information and communication technologies that can be collaboratively supported in different levels including information sharing support and task coordination support. Therefore, the business cooperation in the hospital SPD logistics model contains not only the information sharing in traditional information system, but also workflow optimization of information intelligent delivery and business-oriented collaboration.

### Instant communication technology-based hospital logistics information collaboration

Instant communication combines audio and video communication and files transfer, and provides necessary platform for information sharing and delivering of logistics collaborative BI system. Therefore, the full use of the instant communication technology requires necessary integration of heterogeneous information in the hospital logistics collaborative BI system. In this study, we provided uniformed structural descriptive data for describing information resources in the hospital logistics collaborative BI system with the help of metadata. We accomplished this by establishing metadata level of hospital logistics data warehouse using the Dublin core data sets, and built logistics information integration model. We also attempted to build dynamic matching models of the supply chain and logistics related information (i.e., the information of demand, inventory, as well as distribution) in BI system via analyzing the attribute and operation information of the supply chain in the SPD model.

### SPD workflow optimization-based hospital logistics business collaboration

Workflow is a part of Computer Supported Cooperative Work (CSCW), which plays an important role in researching how to make the groups work cooperatively within the framework of informationization. The SPD workflow is chiefly used to describe the activities of tasks with fixed procedures in the hospital logistics businesses. The core of the SPD workflow optimization is to figure out the “control points” that requires cooperating by mining “up and down streams” and “time rules” in the original workflow. This is to achieve several goals including improved work efficiency, better control process, and effective management of logistics business processes. Therefore, we constructed the up and down streams and time constraint relations of workflow by mining the time data from SPD workflow in the data warehouse, and then built the workflow time constraint relations-based Activity On Edge (AOE) Network. Based on which we figured out the critical paths in SPD workflow net structure through the utilizing of swarm intelligence firefly algorithm and Critical Path Method (CPM).

### Construction of experimental platform

This study intended to construct hospital logistics SPD model-oriented collaborative BI system, by which we realized the data and information sharing between the hospitals, the suppliers and the manufacturers in the hospital logistics supply chain. By using SVM and swarm intelligence firefly algorithm methods, we analyzed the recent years’ basic information database of hospital information system (HIS) and data provided by companies in the logistics supply chain. We also established a hospital logistics SPD model-oriented collaborative BI system that realized such roles as collaboration, data mining, data analysis, as well as application.

The timeliness and collaboration of hospital logistics collaborative BI system in SPD model were improved by the following procedures: (i) through constructing the module of sharing data warehouse, we realized the integration of data marts in hospital logistics management, so as to ensure the data safety and provide services such as basic data process and analysis; (ii) for the extraction of inherent characteristics of data sets, we constructed the module of data preprocessing to reduce the data dimension of collaborative BI system; (iii) by setting up supplier selection optimization module and using the method of SVM, we improved the business processes to meet the demands of supplier evaluation, classification and selection in the hospital logistics SPD model-oriented collaborative BI system; (iv) via establishing inventory optimization module, we were able to solve the inventory optimization problems in hospital logistics warehouse; (v) by creating medical consumables constant distribution module, we could deal with the decision-making processes such as distribution amount, distribution point, distribution lead time, as well as dynamic adjustment of distribution amount; (vi) in order to realize the collaboration of logistics information and information sharing among every parts of the supply chain, we built information collaborative service module; (vii) by building business collaborative service module, we provided the decision-making basis for logistics process optimization in the hospital SPD model. In this study, we chose Windows Vista operating system as the development environment. Other specific requirements are as follows: software structure: J2EE; compile environment of Java language: version JDK 1.5.07; server: open source server developed by Apache Group Jakarta group, version TOMCAT 5.0.28; JSP front-end development platform: Dreamweaver MX 2014; back-end development environment: Eclipse 5.1; database system: Microsoft SQL Server 2008; ETL tools in BI system: PowerCenter; shared data warehouse: Microsoft SQL Server; OLAP server: SAP Essbase; front display tools: Business Object.

## Discussion

With the rapid development of medicine and health services and in the face of ever increasing demand, the use of medical consumables in hospitals is increasing. A hospital, usually, requires multiple varieties of medical consumables with complicated specifications from several suppliers, which results in considerable management difficulties, low management efficiency, as well as unsafe medical activities. We advocate the use of the SPD logistics management model, which have several advantages such as: (i) the procurement information is automatically generated by the system after analyzing the historical data on consumption, inventory level and response time of suppliers. This may largely improve the standardization and automation of procurement; (ii) the inventory of every stage (e.g., the warehouse of center, departments and operating room) is monitored by the logistics management departments, the inventory related variables can be scientifically and coordinately arranged including the ordering point, safe inventory, maximum inventory, and lead time of ordering. This way, we can significantly reduce the cost as well as the inventory risk. (iii) The distribution of consumables is based on inventory optimization, by analyzing the historical consumption habit of clinical departments, the distribution related variables including distribution amount, distribution point, distribution lead time, as well as dynamic adjustment of distribution amount can be properly assigned. By adopting this model, we may reduce the waste of consumables and significantly reduce the workload associated with material management and improve the efficiency of clinical activity.

In China, the informationization of hospital management mainly focuses on the information support of the medical activity management, especially the clinical treatment. The information support for hospital logistics management is relatively weak, which makes it difficult to support the construction and implement of the SPD model. Specific problems were mainly manifested in two aspects from the perspective of management informationization. At first, the data utilization rate was relatively low and the existing information construction hardly met the data mining demands in the complex logistics data. Additionally, the collaboration between information systems was quite lacking. Traditional construction of informationization mainly concentrated on the information electronization in the hospital, rather than the outside links of the supply chain, which ignored the collaborative demands among the hospitals, suppliers and manufacturers. With the development of recent decades, BI system has been widely applied in many fields including manufacturing (20), bank (21), logistics (22) and healthcare (23), etc. In the field of logistics, the application of BI system mainly focused on the intelligent procurement management, intelligent inventory management, as well as the intelligent distribution schedule system (22, 24). While in the field of healthcare, BI system was commonly used in the hospital performance management, hospital cost accounting analysis and medical insurance decision-making, etc. (25, 26). Up to now, very few studies have applied the BI system to hospital logistics management. Therefore, with the implement of medical reform and the adoption of the SPD model, we can construct a collaborative BI system by introducing and applying the idea of BI to hospital logistics. This way, we may realize the business collaboration of hospital logistics management and achieve a few goals such as improving management level and reducing the hospital logistics’ costs.

We may encounter many difficulties in this study, which requires innovating and improving the traditional SPD model and making appropriate implement plans and schedules for the application of BI system according to the actual situations in hospitals. Additionally, given the fact that the study is based on information integration, the successful implementation of this study requires the collaborative participation of internal departments in hospital including the department of information, logistics, nursing, medical and financial. The timely response of external suppliers is the other significant requirement of this study.

## Conclusion

BI transfers various data from enterprises into information or knowledge displayed in a cooperative manner that enterprise managers interested in. Introducing and applying the collaborative BI system to hospital SPD logistics management model may help largely in realizing the business collaboration of hospital logistics management and achieving a few goals such as improving logistics management level, reducing hospital logistics costs, and increasing health service quality as well.

## Ethical considerations

Ethical issues (Including plagiarism, informed consent, misconduct, data fabrication and/or falsification, double publication and/or submission, redundancy, etc.) have been completely observed by the authors.
